# EBI metagenomics in 2016 - an expanding and evolving resource for the analysis and archiving of metagenomic data

**DOI:** 10.1093/nar/gkv1195

**Published:** 2015-11-17

**Authors:** Alex Mitchell, Francois Bucchini, Guy Cochrane, Hubert Denise, Petra ten Hoopen, Matthew Fraser, Sebastien Pesseat, Simon Potter, Maxim Scheremetjew, Peter Sterk, Robert D. Finn

**Affiliations:** European Molecular Biology Laboratory, European Bioinformatics Institute (EMBL-EBI), Wellcome Trust Genome Campus, Hinxton, Cambridgeshire, CB10 1SD, UK

## Abstract

EBI metagenomics (https://www.ebi.ac.uk/metagenomics/) is a freely available hub for the analysis and archiving of metagenomic and metatranscriptomic data. Over the last 2 years, the resource has undergone rapid growth, with an increase of over five-fold in the number of processed samples and consequently represents one of the largest resources of analysed shotgun metagenomes. Here, we report the status of the resource in 2016 and give an overview of new developments. In particular, we describe updates to data content, a complete overhaul of the analysis pipeline, streamlining of data presentation via the website and the development of a new web based tool to compare functional analyses of sequence runs within a study. We also highlight two of the higher profile projects that have been analysed using the resource in the last year: the oceanographic projects Ocean Sampling Day and Tara Oceans.

## INTRODUCTION

The concept of cultivation-independent microbial genomic analysis approaches, which underlie the modern field of metagenomics, were first described over 40 years ago (1,2). These reports were followed by the first actual metagenomic library primary data publication, which described screening and sequencing of recombinant lambda libraries from marine plankton communities for taxonomic characterization (3). However, the term ‘metagenomics’ itself did not appear in publication until 1998, where it was used to describe the collective genomes of soil microflora (4). Since then, metagenomics has gone on to become an umbrella term, which encapsulates a range of study types that use high-throughput DNA sequencing to characterize microbial systems, including whole-genome shotgun (WGS) sequenced metagenomic and metatranscriptomic studies, as well as amplicon-based approaches, targeting specific marker genes. Typically, metagenomic studies involve the analysis of unassembled sequence data, although assembly is becoming more common as the rate of sequencing grows and experimental techniques improve. For example, assembly can be used in an attempt to reconstruct a single dominant microbial genome or to allow full-length genes to be recovered from metagenomic shotgun data.

Metagenomics has become increasingly mainstream in the last decade, partly due to the exposure of high profile projects, such as the Global Ocean Sampling Expedition in 2007 (5) and Human Microbiome Project in 2012 (6). The ability to simultaneously analyse the collective genomes of all microbes within a particular environment provides a powerful insight into microbial community structure, the processes that the community mediates and the complex interactions that may occur. As sequencing costs continue to diminish, the breadth of metagenomic research increases. The approach has now been successfully applied to a wide range of research areas, including agriculture (7,8), bioenergy production (9–11), bioremediation (12), and animal and human health (13–16).

Whilst the take-up of metagenomics is both wide-scale and burgeoning, the analysis of metagenomic sequence data can be particularly challenging and is an increasing bottleneck. One significant problem is the sheer volume of sequencing data generated. For example, a typical Illumina HISEQ 2000 paired-end run, with 150 million forward and reverse 150 nt-long reads, can produce over 50 Gb of data in FASTQ format. Traditional sequence analysis approaches, such as BLAST, are unable to scale with such data volumes (for example, a BLAST search for this size of sequencing run, analysed against the UniProtKB (17) database, would require in the region of 15 billion pairwise sequence comparisons). Substantial compute resources are required, as are specialist analysis algorithms and approaches that are both fast and sensitive, as the majority of organisms present in a metagenomics dataset are not found in typical reference databases. Furthermore, if such data is to have longevity and be reproducible, it must be archived in publicly accessible repositories and described with detailed and accurate contextual data.

EBI Metagenomics (EMG) has been developed as a free-to-use, large-scale analysis platform for metagenomic sequence data. The resource is capable of processing WGS sequenced metagenomic and metatranscriptomic reads, 16S rRNA amplicon data and user-submitted sequence assemblies. Regardless of the data source (metagenomic, metatranscriptomic, amplicon or assembly), EMG provides a standardized analysis workflow, capable of producing rich taxonomic diversity and functional annotations. As a result, analyses can be compared both within and across projects at a broad level, and across different data types (e.g. metagenomic versus metatranscriptomic). Rather than develop an entirely new repository for metagenomic data, EMG has partnered with the European Nucleotide Archive (ENA) (18) to provide a permanent metagenomics data archive and data sharing/publication service. Leveraging the ENA's existing infrastructure and interfaces for data submission ensures that datasets are described with standards-compliant contextual data, and are made available to the scientific community for data mining purposes and for meta-analysis.

In this article, we present the current status of EMG and its data content. We describe recent improvements to the resource, including a revamp of the analysis pipeline that has improved our ability to analyse large-scale projects. We also describe changes to the website, aimed at improving data presentation and discovery and the development of new web tools to enable comparison of functional analysis results within a project.

## REGISTRATION AND DATA SUBMISSION

Users submitting data for analysis by EMG are required to hold a valid ENA submission account that has been registered for use with EBI Metagenomics. This helps ensure that the right contextual data is provided as part of the submission process and that submitted data can be tracked through the ENA's archiving system and identified for analysis. Any users planning to submit confidential pre-publication data for analysis (which may be held privately for up to 2 years) are required to explicitly confirm that they authorize its access by EMG, in accordance with the ENA's data access policies. To streamline the account management process, EMG's interactive web tool (https://www.ebi.ac.uk/metagenomics/submission) has been overhauled to simplify the way users create and register accounts and grant internal EMG access to pre-publication data. In addition, users may use this tool to check or alter the status of their account at any time. Whereas previously, users were required to register with both EMG and ENA, now a single form is provided, with the account management shared across EMG and ENA.

## SUPPORTED SEQUENCING PLATFORMS

EMG continues to provide analysis of sequence data derived from a range of platforms, including Roche 454, Ion Torrent and Illumina (single and paired-end). The resource has also received its first Oxford Nanopore submission, although the sequence quality was insufficient to pass the standard quality control (QC) stage (see below) or to assign annotation through the sequence homology methods used by the pipeline. Nevertheless, we expect to be able to provide meaningful analysis of nanopore long-read sequences as the technology matures and sequence quality improves.

## DATA CONTENT

Analysed data in EMG are now structured into projects, samples and runs. The addition of the run level ensures that EMG mirrors the data object organization in ENA, providing greater consistency between the two resources and a natural relationship between sequence data and the corresponding analysis. In this arrangement, one project contains one or more samples, and each sample can have one or more experiments associated with it (e.g. metagenomic and metatranscriptomic), which can be from individual runs or pooled runs from a sequencing machine.

At the time of writing (September 2015), EMG contains 6705 samples from 132 public and 83 private projects. This represents a growth of over four-fold in the number of projects and five-fold in the number of samples since the first release in 2013 (19). Previously, the resource focused mainly on the analysis of submitted WGS metagenomic data. It thus neglected much of the 16S rRNA amplicon data, which has entered ENA directly or via its INSDC partners. As 16S rRNA data analysis is increasingly used as a diagnostic tool in the human host-associated setting (and often as a precursor to WGS metagenomic analysis) this stance has been reviewed. EMG now provides 16S rRNA amplicon data analysis (even in the absence of WGS metagenomic data—a previous requirement) and existing datasets in ENA can be prioritized for analysis on request.

EMG currently contains analyses for 4037 metagenomes, 2975 16S rRNA amplicon datasets, 389 metatranscriptomes and 67 assemblies. Almost 100 billion nucleotide sequences have now been processed, yielding over 50 billion predicted protein coding sequences (pCDS), ∼18 billion of which have been annotated with functional information.

## ANALYSIS PIPELINE UPDATE

**Figure 1.**
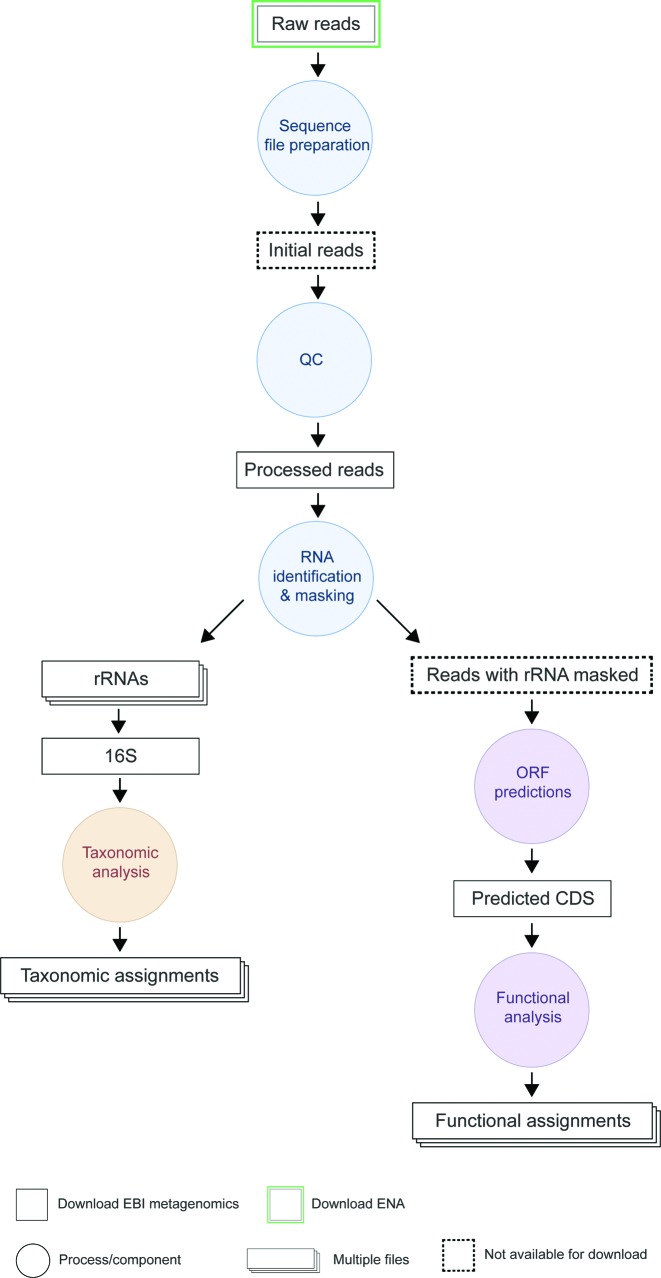
Schematic of the analysis pipeline. Processes/components are indicated as circles and inputs/outputs are represented by rectangles. The structure of pipeline v2.0 is similar to that of v1.0. Following input file preparation and a QC stage (to remove short/low quality reads), the pipeline branches into two parts: one performing taxonomic classification (based on 16S rRNA) and the other providing functional annotation (based on pCDS matches to a subset of the InterPro databases). A full description of the steps, tools and reference libraries used is provided on the EMG website at https://www.ebi.ac.uk/metagenomics/pipelines/2.0.

A new version of the analysis pipeline (v2.0) (Figure 1) was released in March 2015. Since the release of EMG in early 2010 and prior to this update, there had been no major changes to the reference data libraries used by the pipeline—most notably InterPro (20), which was 19 releases behind the most recently available version. Similarly, some of the software algorithms were outdated, with improved versions available. The tools and libraries updated in pipeline v2.0 are summarized in Table 1.

**Table 1.** Updated tools and algorithms used in analysis pipeline version 2.0

Component	Previous version	New version	Function
QIIME/GreenGenes	1.50/12.10	1.90/13.8	16S taxonomic classification
rRNASelector	1.0.0	1.0.1	Identification of rRNA fragments
InterPro/InterProScan	31.0/5-beta	50.0/5.9	Functional annotation

The newer version of rRNASelector uses a more up-to-date version of HMMER (3.1b compared to 3.0), which is substantially faster (∼10-fold). Some of the member databases within InterPro also use HMMER and benefit similarly from an increase in data processing speed following the pipeline update.

The identification of rRNA using rRNASelector represents the last process before the pipeline conceptually branches (Figure 1). With v1.0 of the pipeline, those reads containing rRNA segments were separated from those that lacked an rRNA, with the latter undergoing functional assignments. However, it is feasible for a read to contain both an rRNA and a pCDS. Rather than binning the reads, the rRNAs are therefore masked in v2.0, before being passed on for functional annotation. Another pipeline change was the removal of the clustering and repeat masking steps that formed part of the QC stage in pipeline v1.0. Performance profiling suggested these steps added significant processing bottlenecks, yet contributed little to the overall data quality, nor significantly changed the analysis results. For example, when processing moderately-sized runs (10 Gb Illumina sequencing data, comprising ∼20 million sequences), the clustering step typically took over 12 CPU hours, yet <1% of sequences were merged. An added advantage of removing the sequence clustering step is that pipeline v2.0 is able to directly provide abundance counts.

The pipeline code (wrapping each algorithm) was also substantially refactored as part of the update process to improve performance (e.g. better horizontal scaling), stability (e.g. better error handling) and throughput (e.g. checkpointing and recovery). Furthermore, the development work focused on pipeline modularization, ensuring that both component upgrades and extensions to the pipeline can be more readily achieved in the future. Overall, analysis throughput using the new pipeline is ∼15× faster than with v1.0. Illustrating this increased throughput, almost 3000 runs (comprising over 34 billion nucleotide sequences, ∼⅓ of the content of EMG), have been analysed since the deployment of v2.0 of the pipeline 6 months ago.

Results for projects analysed with both v1.0 and v2.0 of the pipeline are able to co-exist within EMG and, where applicable, data files for both versions are presented on the website. The pipeline version used for analysis is clearly labelled on the project and sample web pages. Users of EMG can request a project be re-analysed with v2.0. Rather than re-analysing all projects, our current objective is to use the increased capacity to focus on adding some of the absent public metagenomic datasets that have entered ENA via other routes, while maintaining the analysis of new projects being submitted to EMG.

## LARGE-SCALE DATA ANALYSIS PROJECTS

Amongst the projects analysed this past year have been two large-scale oceanographic datasets: Ocean Sampling Day (OSD) and Tara Oceans. OSD is a simultaneous sampling for marine micro-organisms in a global network across all continents (21). Tara Oceans (22–26) is a project to map the biodiversity of a wide range of planktonic organisms and their interactions with the surrounding environment. While these projects share a common biome, they have been independently run and analysed by their respective research consortia. However, both projects are united under the EMG, having been archived with ENA (study accessions PRJEB5129 and PRJEB402), described with contextual data compliant to the M2B3 data standard (27) and analysed using v2.0 of the EMG pipeline. The use of a consistent description and analysis approach allows results from the two projects to be compared, identifying common and contrasting functional and taxonomic assignments. They may also be compared to pre-existing marine metagenomes, such as the Global Ocean Sampling Expedition datasets (EMG study accession SRP003580).

EMG provided analysis for the metagenomic component of Ocean Sampling Day data collected in 2014 (EMG study accession ERP009703) and the Global Ocean microbiome subset of the Tara Oceans project, which contains metagenomic sequences, size fractionated for prokaryotes (ERP001736). Whilst roughly equivalent in sample size (150 and 135 samples, respectively), the two projects differ substantially in the amount of sequencing data, with the subset of OSD analysed comprising ∼120 Gb in total data size (∼220 million sequences) and the Tara Oceans subset representing ∼10 Tb of sequence data (∼29 billion sequences).

The EMG analysis predicted ∼180 million protein coding sequences for the OSD2014 project, 68 million of which were annotated with matches to InterPro. For Tara Oceans, over 23 billion protein coding sequences were predicted, over 9 billion of which received InterPro annotation. As far as we are aware, the analysis of these projects is not available through any other metagenomics analysis platform.

**Figure 2.**
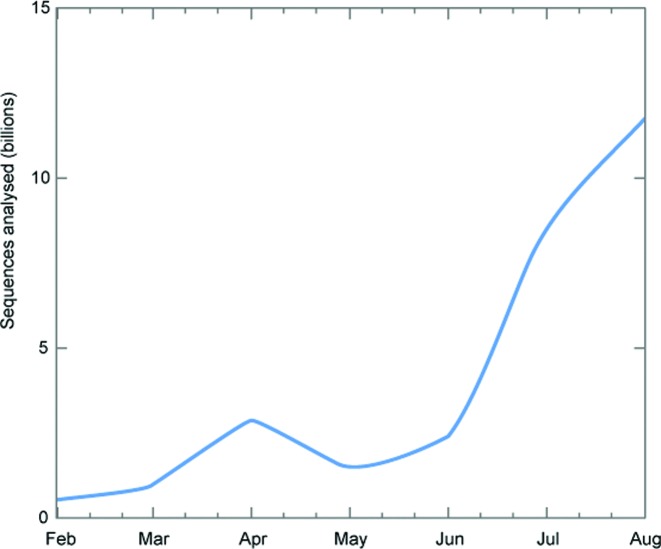
EMG analysis pipeline throughput for the Tara Oceans project, based on analyses completed each month. With relatively static compute resources available, the upward trend is a result of pipeline improvements. The highest value gives an indication of our current expected peak processing capacity.

The prokaryotic microbiome from Tara Oceans is the single largest project to be processed by EMG to date. Some runs were sufficiently large (over 100 Gb) that they stretched the analysis pipeline to its limit, prompting additional refinements to v2.0 to further improve speed. Figure 2 shows the throughput of the pipeline for the Tara analysis over the seven months it took to complete. The lower productivity in May/June is partly due to concentrated efforts aimed at improving the pipeline, the benefits of which can be observed in subsequent months, clearly demonstrating that EMG is well placed to handle future metagenomics projects of this size; if another project the size of Tara Oceans were to be submitted today, it could take a little as 10 weeks to process.

## WEBSITE IMPROVEMENTS

EMG now represents a major (and growing) wealth of taxonomic and functional data pertaining to a broad collection of metagenomics datasets from a myriad of different biomes. As a consequence, EMG no longer a site devoted to data analysis, but is increasingly a place where users come to access existing datasets and to compare their data to flagship projects. To accommodate this additional scope, the EMG website has been extended to increase the discoverability of these data and to facilitate further analysis.

### Discovery by biome

**Figure 3.**
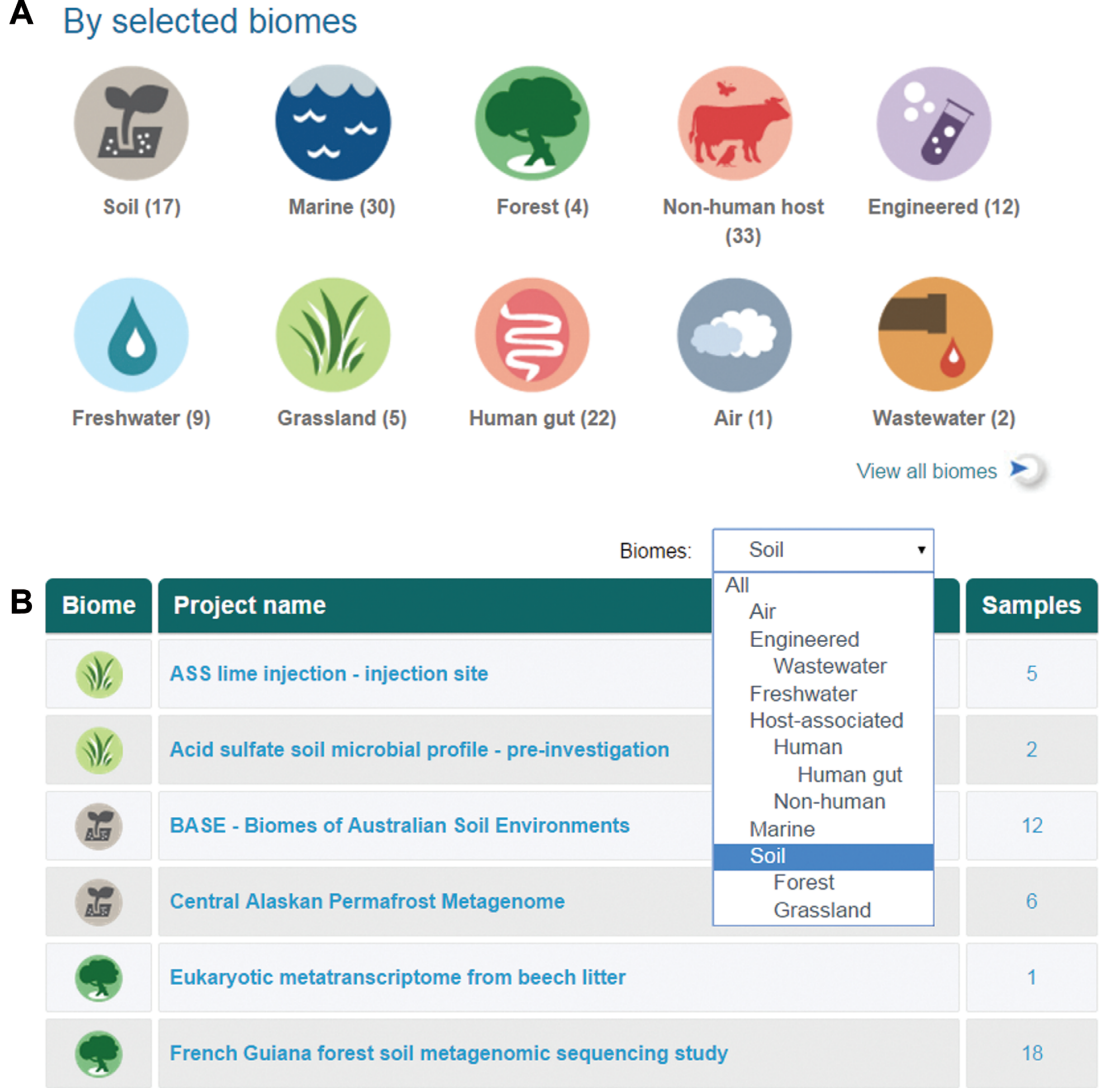
Biome icons and search-by-biome functionality. (**A**) Biomes for projects are indicated by icons on the EMG website. The numbers under the icons represent the number of projects belonging to each biome. (**B**) The biome filter allows users to select a biome of interest (for example marine) and returns matching projects.

EMG contains data from a wide range of different environmental biomes. A ‘search by biome’ feature has been developed, to aid data exploration and allow identification of projects from the same environmental category (Figure 3). The source biome for projects is now indicated by a specific icon on the web pages. The biome data is manually curated in EMG, using the classification scheme developed by the Genomes OnLine Database (https://gold.jgi.doe.gov/) (28). The biome classification is arranged as a hierarchy—for example, the soil biome can be subclassified into grassland or forest soil.

### Analysis results comparisons

**Figure 4.**
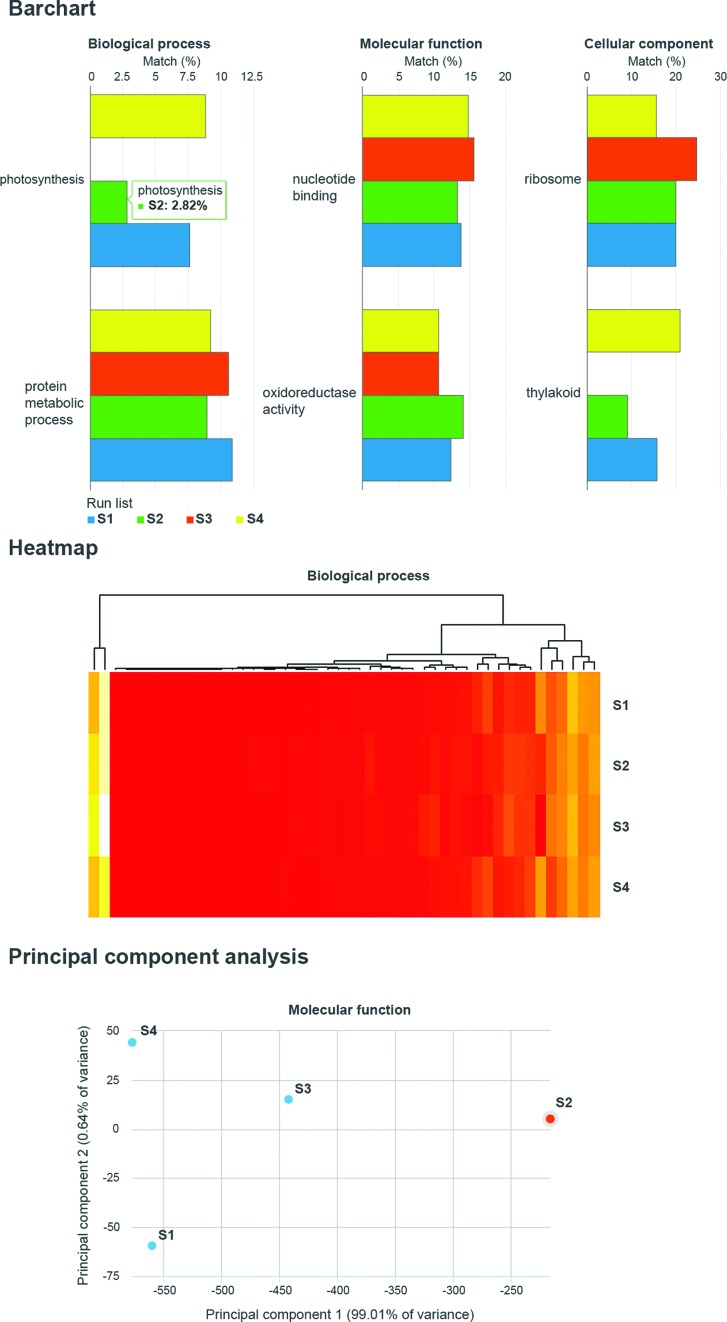
Examples of different data visualizations available via the online comparison tool. The high level GO terms assigned to runs within a project can be compared to each other via bar charts, stacked columns, PCA plots and heatmaps.

EMG has also deployed a web tool that allows the direct comparison of Gene Ontology (GO) (29) terms (summarized using a GO slim developed for metagenomic data (http://www.geneontology.org/ontology/subsets/goslim_metagenomics.obo)) that have been assigned to runs within a project. The tool (which is accessed by clicking on the ‘Comparison tool’ tab on the main web page) presents users with a list of projects with data suitable for comparison (i.e. amplicon datasets are excluded). Selecting a project brings up a set of runs from within the project, which can be compared using the tool. The comparison results are displayed as bar charts, stacked columns, heatmaps and principle component analyses (examples of three of these views are shown in Figure 4). The visualizations can be exported in PDF, PNG and SVG formats. We aim to expand the functionality of this tool as development of the EMG's web interface continues, adding support for full GO term listings, InterPro annotations and taxonomic assignments.

### Data compression

With the growth in project size, some of the files provided via the website download section were substantial (for example, for the larger Tara samples, post-QC sequence files and InterPro annotation files were around 50 and 35 Gb, respectively). To mitigate some of the problems associated with large file download, such as timeout while downloading, we have implemented additional pipeline steps to break these files into compressed (gzipped) chunks. In addition, while data submissions in the CRAM compression format are already accepted, we are also exploring compression approaches both for internal data processing purposes and public data presentation.

### Summary files

**Figure 5.**
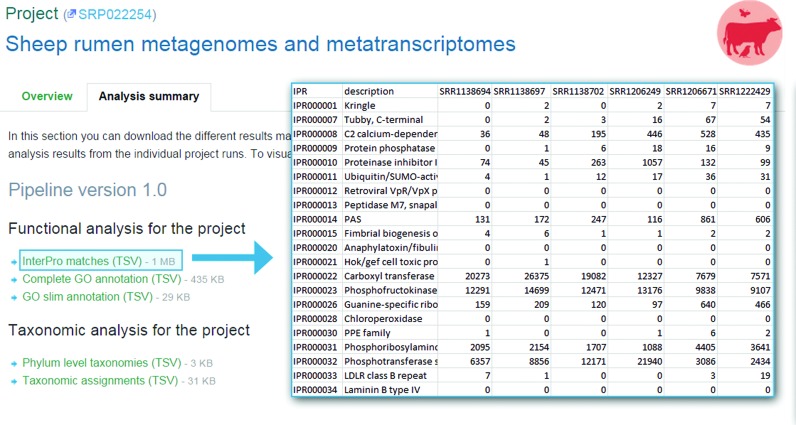
Analysis summary files at the project level are available to download in TSV format. In this example, pCDS matching InterPro entries (rows) for all runs in a project (columns) are provided.

In order to provide users with a summary of analysis results for a particular study, we now aggregate the functional and taxonomic annotations across all runs for each project and make these summary files available (in TSV format) via the EMG website. These files can be found on the project pages under the ‘Analysis summary’ tab (Figure 5). The study summary data allows users to explore datasets at a high level of detail, providing a first step for in-depth metagenome exploration.

## EXISTING PUBLICLY AVAILABLE METAGENOMICS DATASETS

As mentioned above, there are many publicly available metagenomics datasets that are yet to be analysed by the EMG: we recently surveyed the status of ENA and revealed that there are over 900 metagenomics projects that have been deposited in the ENA since 2006, yet do not appear in EMG. With the increased capacity of the EMG pipeline, we have started the process of analysing some of these datasets. Nevertheless, one of the limiting factors in promoting these projects for EMG analysis is often the low quality of contextual information provided by the submitter. To investigate whether this situation could be improved by involving the scientific research community, metagenomic projects were selected as use cases for a ‘Sample Record Annotation Workshop’, organized jointly between the ENA and EMG teams, and external scientists. This intensive annotation jamboree, held in December 2014, aimed at enriching the contextual information of selected metagenomic samples, thereby bringing them up to EMG's standard for processing. Over 1900 samples from 13 publicly available metagenomic projects were annotated as part of the workshop. Primary sequence data from almost 1700 samples from eight of these projects passed the EMG read data QC, and their metagenomic analysis has been published on the website so that results can be discovered, downloaded and compared.

## DISCUSSION

The data content of EMG has expanded considerably over the last 2 years, with 4148 new WGS metagenomic datasets and pipeline developments helping to ensure the resource is well-placed to cope with the growth in demand. In terms of publicly available datasets, EMG now contains comparable numbers of WGS metagenomic samples to portals such as MG-RAST (30) (6231 publicly available metagenomic samples), IMG/MG (31) (3193 metagenomic samples) and iMicrobe (http://imicrobe.us) (2629 metagenomic and amplicon samples). Whilst analysis of some projects may overlap between these portals, they each contain unique datasets and features, and thus offer complementary analyses of metagenomic data. Ensuring a more systematic organization of metaegnomics datasets and their analysis across all platforms is a key challenge for the community in the future.

The quality of associated contextual data is particularly important to the data discovery process, as it is directly proportional to the discoverability and usability of analysed projects. International efforts, such as the INSDC, Genomic Standards Consortium (GSC, http://gensc.org/) and Global Microbial Identifier (GMI, http://www.globalmicrobialidentifier.org/), focus intensively on development of contextual data standards that allow consistent description of sequence data. ENA and EMG are actively involved, especially in development of contextual information checklists for capturing provenance of sequenced and analysed samples. The growing list of ENA sample contextual data checklists currently contains 15 GSC MIxS environments, two marine environments developed for support of the marine enterprises Tara Oceans and OSD, and four microbial pathogen-related checklists. These checklists are developed as standards evolve over time. Other metagenomics resources, such as MG-RAST and IMG/M, have also recognized the fundamental importance of contextual information and actively support efforts of contextual data standardization.

The recent improvements to EMG's analysis pipeline have increased the analysis capacity of the resource. As a result, there is now an opportunity to extend its remit. Nevertheless, even with EMG's increase in analysis capacity, it is important that we continue to evaluate and balance its analysis priorities. With this in mind, we have chosen to prioritize both new submissions and publicly available datasets that have not yet received analysis. As described above, there are a large number of such projects deposited with ENA. We are currently ranking these projects for analysis based on a range of criteria, such as quality of contextual information, size (the number of nucleotide sequences per run file, the number of samples within the project and so on) and whether or not the project is associated with a scientific publication. In particular, we are prioritizing marine and human host-associated projects. These will provide a steady stream of additional analyses, supplementing user-submitted projects and expanding the data content of EMG.

As the data content grows, EMG increasingly becomes a platform for data discovery. To support this process, we have made a series of user-interface improvements, including classification of projects by biome, processing of results files for easier download and provision of project level summary files. We will continue the interface improvement process, as projects become larger and in response to feedback from our users (for example, providing APIs for data discovery and download). As metagenomics research becomes less correlative and more predictive, it will become increasingly important that EMG allows users to slice across datasets to compare and contrast analysis results.

One particularly important area for EMG is the taxonomic analysis of non-prokaryotic organisms. As part of our collaborative analysis work performed on the OSD project (which was not size fractionated prior to sequencing), we performed a series of preliminary taxonomic investigations beyond the scope of the standard analysis pipeline. These revealed a rich array of eukaryotes and viruses, in addition to prokaryotes, within the samples. We were also able to detect interesting relationships between some of the organisms, for example a correlation between certain viruses and their hosts. Developing a generic, scalable and accurate taxonomic analysis component for all micro-organisms is essential for a more complete understanding of community structure.
